# Endocrine, metabolic, and hepatic dysfunction in patients with COVID-19 pneumonia with severe and critically ill status

**DOI:** 10.1186/s43168-022-00118-w

**Published:** 2022-03-10

**Authors:** Ahmed Elesdoudy

**Affiliations:** Chest Diseases and Tuberculosis Department, Faculty of Medicine, Menoufia University, Yassin Abdelghaffar street, Shebin Alkom, Menoufia Egypt

**Keywords:** COVID-19, Electrolytes, Hepatic, Pancreatic, Thyroid

## Abstract

**Background:**

Management of endocrine and hepatic disorders is very important for better management of patient with COVID-19 infections. Hepatic and endocrine dysfunction needs clinical assessment, continued monitoring, and specific treatment. It is important to understand clearly the potential mechanisms causing hepatic and endocrine injury

**Objectives:**

To assess the endocrine and hepatic dysfunctions in patient with COVID-19 pneumonia with severe and critically ill status.

**Methods:**

This retrospective analytical study was performed on 75 patients admitted to intensive care or high dependency units (ICU/HDU) in Obaidullah Hospital, Ministry of Health, United Arab Emirates. All patients were subjected to the following on admission: history taking, general and local examinations, routine laboratory studies (CBC, liver function tests, and kidney function tests), and other laboratory tests: C-reactive protein (CRP), D-dimer, Chest X-ray. Endocrine and hepatic function tests and other laboratory studies are repeated daily to show the progress of endocrine and hepatic functions.

**Results:**

The age range of the studied group was between 35 years and 107 years with mean age 59.98 ± 12.88. The sex distribution was (42 male, 56%) and (33 female, 44%). The associated comorbidities were 4 patients had bronchial asthma (5.3%), 12 patients had chronic kidney disease (16%), 30 patients had diabetes mellitus (40%), 26 patients had hypertension (34.7%), 2 patient had hypothyroidism (2.7%), 12 patient had ischemic heart disease (16%), and 21 patients did not have any comorbidities (28%). There was significant difference between glucose level, thyroid stimulating hormone and free t4 before and after admission (*P* values are 0.001, 0.04, and 0.023 respectively). There was significant difference between serum electrolytes before and after admission (*P* value = 0.001). There was significant difference between liver function test before and after admission (*P* values are 0.004, 0.001, and 0.001)

**Conclusions:**

Hepatic, pancreatic, thyroid functions, and electrolytes are affected by COVID-19 infection. These could act as surrogate biomarkers for better management of hepatic, pancreatic, thyroid functions and electrolytes disturbances in patients with COVID-19 infection.

## Introduction

Since December 2019, the new coronavirus (2019-nCoV or COVID-19) was detected in Wuhan, China. The COVID-19 has then rapidly spread worldwide [1]. Due to the absence of an efficient treatment for SARS-COV-2, social distancing, and hand hygiene seems the most effective modes to decelerate this pandemic spread. For ensuring the same, governments all over the world have imposed nation-wide lockdown. Because of these prevailing events, managing the preexisting medical disorders, including endocrine disorders, may appear on the horizon. With about 10% of the general populations are affected by diabetes mellitus [2], about 5% by thyroid hypo-function, 0.2–1.3% by thyroid hyper-function [3], managing of the endocrine dysfunctions is very important in patients with COVID-19 infections.

Despite the earlier clinical trials, especially those performed in the USA, China, and Italy, have marked the main clinical manifestations including fever, cough, fatigue and breath shortness, the subsequent studies highlighted the extra-pulmonary disease manifestations. These trials marked that after the adult respiratory distress syndrome corona-virus 2 (SARS-CoV-2), a progressive disease and even the infectious virus itself may progress to involve many systems in the body and multi-organ failure. The liver is the main organ for detoxifying and metabolizing of toxic materials and drugs, and the maintenance of hepatic functions is important for engaging all available treatment modes in the management of COVID-19. Hepatic dysfunction needs clinical assessment, continued monitoring, and specific treatment. For a better management of COVID-19, it is important to understand clearly the potential mechanisms causing in hepatic injury [4].

The aim of the study was to assess the endocrine and hepatic dysfunctions in patient with COVID-19 pneumonia with severe and critically ill status

## Methods

This is a retrospective analytical study have been done in Ibrahim Bin Hamad Obaidullah Hospital, Ministry of Health, United Arab Emirates. This retrospective study was performed on 75 patients who already have been admitted to intensive care or high dependency units (ICU/HDU) from January 2021 to May 2021. The data were collected retrospectively from the hospital filing system. All patients were subjected to the following on admission: careful history taking, general and local examinations, routine laboratory studies (CBC, liver function tests, and kidney function tests), and other laboratory tests: C-reactive protein (CRP), D-dimer, and chest X-ray. Endocrine and hepatic function tests and other laboratory studies were repeated daily to show the progress of endocrine and hepatic functions. All hepatotoxic medication stopped. The laboratory tests were done using reagents from Siemens Company, Germany. The reagent for electrolytes was Quiklyte. The reagents for liver function tests were DBI, ALTI, ASTI, ALB, and TP. The reagents for kidney function were UREA and CRE2. The device used for CBC was ADVIA 560, Hematology System, Siemens Company, Germany. Clinical inclusion criteria: adult in-patients diagnosed with severe and critically ill COVID-19 infection. Severe disease: patients with respiratory rate > 30 breaths per minute, SpO2 < 94% on room air (patients with chronic hypoxia, a decrease from baseline of > 3%), ratio of arterial partial pressure of oxygen to fraction of inspired oxygen (PaO2/FiO2) < 300 mmHg, or lung infiltrates > 50%. Critical disease: patients with respiratory failure, septic shock, and/or multi-organ failure. Clinical exclusion criteria: other adult/pediatrics patients diagnosed with asymptomatic, mild, and moderate COVID-19 infection.

### Statistical analysis

Results were collected, tabulated, and statistically analyzed by IBM personal computer and statistical package SPSS for windows version 21, EpiCalc and Microsoft Excel. Two types of statistics were done: descriptive statistics: e.g., percentage (%), mean *(x)*, and standard deviation (SD); and analytic statistics: e.g., Student’s *t* test which is a test of significance used for comparison between two groups having normally distributed quantitative variables and Mann-Whitney test which is a test of significance used for comparison between two groups having quantitative variables which are not normally distributed. *P* value < 0.05 was considered statistically significant (S).

## Results

This study showed that the age range of the studied group was between 35 years and 107 years with mean age 59.98 ± 12.88. The sex distribution of the studied group was group (42 male, 56%) and (33 female, 44%). The outcome of the studied group was 52 patients deteriorated (69.3%), 23 patients improved (30.6%).This study showed that the associated comorbidities of the group were 4 patients had bronchial asthma (5.3%), 12 patients had chronic kidney disease (CKD) (16%), 30 patients had diabetes mellitus (DM) (40%), 26 patients had hypertension (HTN) (34.7%), 2 patient had hypothyroidism (2.7%), 12 patient had ishemic heart disease (IHD) (16%), and 21 patients did not have any comorbidities (28%). This study showed the progress of patients’ symptoms where the shortness of breath (SOB) deteriorated in 52 patients (69.3%), improved in 23 patients (30.6%).The cough symptom deteriorated in 52 patients (69.3%), improved in 23 patients (30.6%). The progress of patients’ radiology (chest X-ray) where it is deteriorated in 52 patients (69.3%), improved in 23 patients (30.6%).The length of hospitalization was more prolonged in patient with comorbidities than patients without comorbidities (35.65 days versus 22.32 days) (Table 1). There is statistical significant impairment in glucose level, thyroid stimulating hormone (TSH) and free t4 before and after admission (*P* values are 0.001, 0.04, and 0.023, respectively). It also showed that there is significant statistical impairment in serum electrolytes (Na, K, Ca, Mg, and phosphorous) before and after admission (*P* value = 0.001). Regarding liver functions, there is statistical significant impairment in liver function test (ALT, AST, albumin) before and after admission (*P* values are 0.004, 0.001, and 0.001) (Table 2). The study also showed that there is significant statistical impairment in vital signs (temperature, pulse, respiratory rate) (*P* value 0.001), laboratory data (WBCs, lymphocytes, CRP, D-dimer) (*P* value 0.001) on and after admission (Table 3).

**Table 1:** Age, sex distribution, outcome, comorbidities, symptoms, and chest X-ray of the studied group

**Age**		
Mean ± SD	59.98 ± 12.88	
Median	61.5	
Range	35.00–107.00	
**Sex**	No	%
Male	42	56
Female	33	44
**Outcome**	No	%
Deteriorated	52	69.3
Improved	23	30.6
**Comorbidities**	No	%
Asthma	4	5.3
Chronic kidney disease (CKD)	12	16
Diabetes mellitus (DM)	30	40
Hypertension (HTN)	26	34.7
Hypothyroidism	2	2.7
Ischemic heart disease (IHD)	12	16
None	21	28
**Symptoms**	No	%
**SOB**		
Deteriorated	52	69.3
Improved	23	30.6
**Cough**		
Deteriorated	52	69.3
Improved	23	30.6
**Chest X-ray after admission**	No	%
Deteriorated	52	69.3
Improved	23	30.6
**Length of hospitalization**		
Patients with comorbidities	35.65 days	
Patients without comorbidities	22.32 days	

*SD* = standard deviation

**Table 2:** Endocrine parameters, electrolytes, and liver functions before and after admission

	On admission	After admission	Student’s *t* test	*P* value
	Mean ± SD	Mean ± SD		
Glucose (mmol/L)	8.97 ± 2.15	13.27 ± 3.81	− 7.267	0.001^a^
TSH (μIU/mL)	1.97 ± 1.60	2.27 ± 1.56	− 2.06	0.04^a^
free t4 (pmol/L)	14.73 ± 2.98	15.46 ± 2.24	− 2.35	0.023^a^
NA (mmol/L)	143.89 ± 5.54	138.52 ± 3.64	− 8.192	0.001^a^
K (mmol/L)	4.76 ± 1.11	4.18 ± 0.26	− 4.47	0.001^a^
Ca (mg/dL)	2.31 ± 0.16	1.91 ± 0.14	19.36	0.001^a^
Mg (mmol/L)	1.01 ± 0.07	0.85 ± 0.11	13.79	0.001^a^
Phosphorus (mmol/L)	1.06 ± 0.23	0.87 ± 0.17	12.24	0.001^a^
Albumin **(g/L)**	33.20 ± 6.73	19.18 ± 5.07	15.732	0.001^a^
	Mean ± SD	Mean ± SD	Mann-Whitney test	*P* value
ALT(IU/L)	61.49 ± 58.91	111.37 ± 100.69	− 3.015	0.004^a^
AST(IU/L)	40.53 ± 17.89	79.82 ± 73.39	− 3.768	0.001^a^

*TSH* = thyroid stimulating hormone, *t4* = tetra iodothyronin, *NA* = serum sodium, *K* = serum potassium, *Ca* = serum calcium, Mg = serum magnesium, *ALT* = alanine aminotransferase, *AST* = aspartate aminotransferase***,*** *SD* = standard deviation

^a^Significant difference

**Table 3:** Vital signs and laboratory studies of the studied group

Paired samples statistics	On admission	After admission	Students’ *t* test	*P* value
	Mean ± SD	Mean ± SD		
Temp (°C)	37.633 ± 0.855	38.008 ± 0.886	− 4.504	0.001^a^
Pulse (beat/min)	73.168 ± 12.419	78.714 ± 16.573	− 3.739	0.001^a^
RR (breath/min)	21.050 ± 6.421	24.196 ± 7.881	− 4.287	0.001^a^
WBCs/μL	6249.546 ± 2440.329	5626.818 ± 2343.335	4.104	0.001^a^
Lymphocytes/μL	2.083 ± 0.801	1.829 ± 0.740	3.709	0.001^a^
CRP (mg/L)	43.255 ± 56.209	52.885 ± 47.854	− 2.621	0.009^a^
D-dimer (μg/mL)	2.956 ± 4.097	1.880 ± 1.611	3.750	0.001^a^

*SD* = standard deviation, *WBCs* = white blood cells, *CRP* = C-reactive protein

^a^Significant

## Discussion

This study showed that the age range of the studied group was between 35 years and 107 years with mean age (59.98 ± 12.88). The sex distribution of the studied group was (42 male, 56%) and (33 female, 44%).

The outcome of the studied group was 52 patients deteriorated (69.3%) and 23 patients improved (30.6%) respectively.

This in agreement with the study done by James Lok et al. [5]. It stated that abnormal liver function is associated with a negative outcome among those hospitalized with COVID-19. The cause for this association is unclear, but correlation between abnormal liver function and higher serum levels of acute phase proteins suggest that dysregulation of the immune system in response to SARS-CoV-2 may be contributory

The associated comorbidities of the studied groups were 4 patients had bronchial asthma (5.3%), 12 patients had chronic kidney disease (CKD) (16%), 30 patients had diabetes mellitus (DM) (40%), 26 patients had hypertension (HTN) (34.7%), 2 patient had hypothyroidism (2.7%), 12 patient had ischemic heart disease (IHD) (16%), and 21 patients did not have any comorbidities (28%).

A large-scale trial of the Centers for Disease Control and Prevention of the United States [6] demonstrated that 78% of the patients with COVID-19 in ICUs had diabetes, cardio-vascular diseases including hypertension or chronic pulmonary disease. A trial carried out by the Chinese Centre for Disease Control and Prevention [7] involving 72,314 patients (admitted to hospital and outpatient) showed a global death rate of 2.3% (1023 deaths in 44,672 positive COVID-19 subjects) and in patients having diabetes it 7.3%. Another report from China [8] involving 1590 in-patients analyzed admission to ICU, invasive ventilation, or death, and after adjustment of smoking status and age. It showed that diabetes increased significantly the severity risk (hazard ratio 1.59, 95% CI 1.03–2.45); in that study, 34.6% of cases with severe disease had diabetes in contrast to 14.3% in non-severe cases.

This study showed the progress of patients’ symptoms where the shortness of breath (SOB) deteriorated in 52 patients (69.3%), improved in 23 patients (30.6%). The cough symptom deteriorated in 52 patients (69.3%), improved in 23 patients (30.6%). The progress of patients’ radiology (chest X-ray) where it is deteriorated in 52 patients (69.3%), improved in 23 patients (30.6%)

This result showed the effect of the already present comorbidities and endocrine and hepatic dysfunction on the progress of the patient symptoms and radiology. Most patients deteriorate after admission due to the effect of these factors.

The length of hospitalization was more prolonged in patient with comorbidities than patients without comorbidities (35.65 days versus 22.32 days).

The study showed that there was statistical significant difference between glucose level, thyroid stimulating hormone (TSH), and free t4 before and after admission.

Huang et al. [9] showed low normal T3, T4, and TSH in COVID-19 without clinically apparent hypothyroidism. The study of Wan G W et al. [10] showed low T3 and TSH in COVID-19 patients in contrast to controls. Brancatella A et al. [11] reported post-COVID-19 subacute thyroiditis from Italy. Ippolito S et al. [12] described thyrotoxicosis in a patient during the in-hospital stay. A recent study by Muller I et al. [13] has highlighted the prevalence of atypical subacute thyroiditis in COVID-19 patients receiving high-intensity care. Of 8 patients who were followed, 2 developed hypothyroidism and 6 had suppressed TSH suggesting long-term effects of COVID-19 on thyroid functions. They were negative for thyroid antibodies. A cell-mediated immune mechanism was suggested by the scientists to explain these findings.

Wang et al. [14] showed pancreatic injury in 17% of 52 COVID-19 patients with elevated serum amylase and lipase levels and two third had abnormal blood glucose levels. Baltar et al [15] reported isolated elevated lipase in COVID-19. Hadi et al. [16] found 2 cases with severe acute pancreatitis in three family members with COVID-19 infection. Further, drugs being used in COVID-19 can result in acute pancreatitis.

A study by Li et al [17] showed that 6.4% of 658 patients presented with ketoacidosis without clear cause. A large-scale database, CoviDIAB Project, by Rubino F et al. [18] has been established for collecting information from patients with COVID-19 and high blood sugar. The inflammatory milieu and elevated levels of cytokines in diabetes may cause severe disease and ARDS. The bidirectional relationship between diabetes and COVID-19 needs further investigations. Bello-Chavolla OY et al. [19] reported that diabetes remains a leading risk factor in severe COVID-19 disease and a predictor for morbidity and mortality. Lacobellis et al. [20] found that admission hyperglycemia was the best predictor of SARS CoV-2 radiological findings.

The study showed that there was significant statistical difference between serum electrolytes (Na, K, Ca, Mg, and phosphorous) before and after admission.

Hugo De Carvalho et al. [21] stated that hyponatremia was prevalent in patient cases than in controls, as well as hypokalemia and hypochloremia. According to the findings of the multivariate study, hyponatremia, and hypokalemia were associated with COVID-19 in patients cases globally, with an adjusted odds ratio of 1.89 [95% CI 1.24–2.89] for hyponatremia and 1.76 [95% CI 1.20–2.60] for hypokalemia. Hyponatremia and hypokalemia independently occurred in association with COVID-19 infection in adult patients who visited the ER, and might act as markers for the physician in the emergency departments in suspected patients with COVID-19.

H. Sarvazad et al. [22] stated that, from all the involved 134 subjects, 49.1% hyperglycemia, 38% hyponatremia, 7.3% hypokalemia, and 32% hypomagnesemia were noted. There was a significant statistical difference between the outpatient and ICU groups regarding FBS, Na^+^, and Mg^2+^. Hyperglycemia and electrolyte disturbances in patients with COVID-19 are feasible; so, the measurement and follow-up of these cases can be efficient in the management and prevention of the severe sequelae of the disease.

The study also showed that there was statistical significant difference between liver function test (ALT, AST, albumin) before and after admission

In the study of James Lok et al. [5] between 16th March 2020 and 30th April 2020, 343 cases were admitted to the acute medical team at Kingston Hospital. Excluding subjects with a history of hepatic disease, 299 cases had hepatic function tests done with abnormal functions found in 44.8% of cases. Disturbance of hepatic function coincided with more requirements for ventilation, admission to high dependency unit or intensive care and prolonged in hospital stay.

Zeng-hong Wu et al. [23] reported a significant relation between hepatic malfunction and mortality of patients with COVID-19 with a pooled OR of 1.98 (95% CI 1.39–2.82). There was a significant difference between AST and severity of COVID-19 with a pooled OR of 4.48 (95% CI 3.24–7.21), and a pooled WMD of 3.35 (95% CI 2.07 to 4.64). In addition, there was a significant difference between TBIL and severity of COVID-19, with a pooled OR of 1.91 (95% CI 1.40–2.60), and with a pooled WMD of 1.18 (95% CI 0.78 to 1.58).

The study also showed that there is significant statistical difference in vital signs (temperature, pulse, respiratory rate) (*P* value 0.001), laboratory data (WBCs, lymphocytes, CRP, D-dimer) (*P* value 0.001) on and after admission (Table 3).

The study showed that there is deterioration in vital signs and laboratory data post admission mostly due to the effect of associated comorbidities and endocrine and hepatic dysfunction.

## Conclusion

This retrospective study found that pancreatic, thyroid functions and electrolytes are affected by COVID-19 infection. These could act as markers for better management of pancreatic, thyroid functions, and electrolytes disturbances in patients with COVID-19 infection.

Severity of COVID-19 patients was significantly associated with liver malfunction. COVID-19 patients with severe disease have elevated serum ALT, AST levels, and decrease in serum albumin level. The findings of the study put a basis for good management of the liver clinically for COVID-19 patients.

## Data Availability

The data that support the findings of this study are available from Ibrahim Bin Hamad Hospital, UAE, but restrictions apply to the availability of these data, which were used under license for the current study, and so are not publicly available. Data are however available from the authors upon reasonable request and with permission of Ibrahim Bin Hamad Hospital, UAE

## Abbreviations

CBC: Complete blood count
SARS: Severe acute respiratory syndrome
DBI: Direct bilirubin reagent for Siemens devices
ALTI: Alanine aminotransferase reagent for Siemens devices
AST: Aspartate aminotransferase reagent for Siemens devices
TP: Total protein reagent for Siemens devices
Na: Serum sodium
K: Serum potassium
Ca: Serum calcium
Mg: Serum magnesium
ICU: Intensive care unit
TSH: Thyroid stimulating hormones
